# A magneto-optical biochip for rapid assay based on the Cotton–Mouton effect of γ-Fe_2_O_3_@Au core/shell nanoparticles

**DOI:** 10.1186/s12951-021-01030-z

**Published:** 2021-10-01

**Authors:** Kuen-Lin Chen, Zih-Yan Yang, Chin-Wei Lin

**Affiliations:** 1 Institute of Nanoscience, National Chung Hsing University, 250, Kuo Kuang Rd., Taichung, 402 Taiwan, ROC; 2 Department of Physics, National Chung Hsing University, Taichung, Taiwan; 3 Graduate Institute of Applied Physics, National Taiwan University, Taipei, Taiwan

**Keywords:** Biochip, Cotton–Mouton (CM) effect, Nanoparticles, SARS-CoV-2, COVID-19

## Abstract

**Background:**

In the past decades, different diseases and viruses, such as Ebola, MERS and COVID-19, impacted the human society and caused huge cost in different fields. With the increasing threat from the new or unknown diseases, the demand of rapid and sensitive assay method is more and more urgent.

**Results:**

In this work, we developed a magneto-optical biochip based on the Cotton–Mouton effect of γ-Fe_2_O_3_@Au core/shell magnetic nanoparticles. We performed a proof-of-concept experiment for the detection of the spike glycoprotein S of severe acute respiratory syndrome coronavirus 2 (SARS-CoV-2). The assay was achieved by measuring the magneto-optical Cotton–Mouton effect of the biochip. This magneto-optical biochip can not only be used to detect SARS-CoV-2 but also can be easily modified for other diseases assay.

**Conclusion:**

The assay process is simple and the whole testing time takes only 50 min including 3 min for the CM rotation measurement. The detection limit of our method for the spike glycoprotein S of SARS-CoV-2 is estimated as low as 0.27 ng/mL (3.4 pM).

**Graphic abstract:**

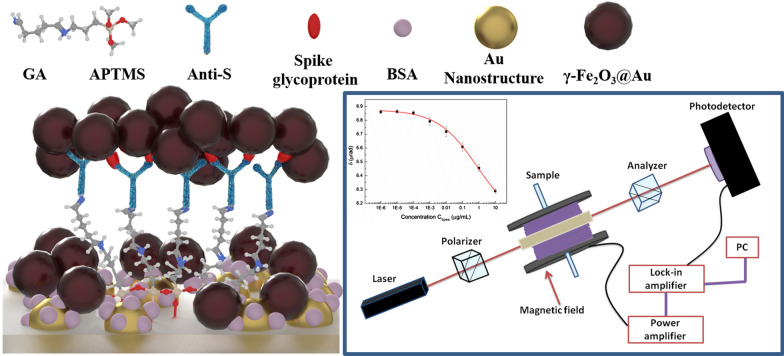

**Supplementary Information:**

The online version contains supplementary material available at 10.1186/s12951-021-01030-z.

## Highlights

1. We developed a biochip for rapid assay based on the magneto-optical Cotton–Mouton (CM) effect of γ-Fe_2_O_3_@Au core/shell magnetic nanoparticles.
2. The easy and quick assay for detection of the spike glycoprotein S of SARS-CoV-2 was demonstrated, and whole process takes approximately 50 min including 3 min for the CM rotation measurement with the detection limit of 0.27 ng/mL (3.4 pM).
3. This magneto-optical biochip we proposed can be easily modified to use as assays for other diseases.

**Supplementary Information:**

The online version contains supplementary material available at 10.1186/s12951-021-01030-z.

## Introduction

Since the outbreak at the end of 2019, coronavirus disease 2019 (COVID-19) has caused a global pandemic. The health and safety of individuals worldwide have been seriously threatened. It was quickly confirmed as a new type of coronavirus and named severe acute respiratory syndrome coronavirus 2 (SARS-CoV-2) by the International Committee of Taxonomy of Viruses [1–3]. As of June 2021, there have been about 0.17 billion confirmed cases and 3.8 million deaths globally, and these numbers continue to increase. Because SARS-CoV-2 is a totally new virus that we never met before, it seriously strikes the medical and public health system all over the world. From the fight experience with COVID-19, we can realize that a sensitive and rapid disease assay method is very important to hinder the disease spread at the beginning of pandemic. To be inspired by the COVID-19 pandemic, we developed a biochip for the rapid disease assay based on the magneto-optical (MO) Cotton–Mouton (CM) effect of γ-Fe_2_O_3_@Au core/shell magnetic nanoparticles (MNPs).

It is well known that MNPs have been widely used in many fields and also play an important role in biomedical applications [4]. With respect to other nanoparticles, MNPs possess the unique magnetic property which increases the applicability of MNPs in the medical field, such as MRI contrast agent [5], drug delivery [6], cancer therapy [7], hyperthermia [8], magneto-photothermal therapy [9] and antibacterial property [10]. Moreover, MNPs have also been used in bio-detection. Biosensor utilizing γ-Fe_2_O_3_ nanoparticles as a detection medium has been reported by Maciel et al. [11]. They used γ- Fe_2_O_3_ as an adsorbent of DNA. The PCR and electrophoresis assays were applied to detected the nanocomposite of the γ-Fe_2_O_3_ and DNA. The magnetism also endows some special characteristics to the MNPs, such as magneto-optical (MO) property. When the magnetic field is applied to the MNPs suspension, the MNPs will be aligned with the direction of the magnetic field. In other word, the magnetic field changes the microstructure of the MNPs suspension and make them anisotropic. When propagating through the anisotropic medium, the velocity of the light will be influenced by its polarization which induces the phenomenon of birefringence. Namely, the optical properties of the medium can be used to exam the status of the MNPs. If the applied magnetic field is perpendicular to the propagation of light, the phenomenon is called CM effect. The CM effect has been applied to the field of biomedicine. Cooperated with the Brownian relaxation, CM effect can be used to detect IgG, IgM and other different biomarkers [12–14]. Besides, Koralewski et al. have found that CM constant is related to iron concentration and diameter of the MNP [15]. However, the medical application based on Cotton-Mouton effect is still relatively rare. Cotton-Mouton effect of most of the biological proteins is very hard to be observed with the small magnetic field (< 150 gauss). Cotton-Mouton measurement for specific target could have a better signal-to-noise ratio due to the low the background noise from other biological composition.

In this work, we demonstrated a magneto-optical biochip and utilized the MO CM effect to detect the spike glycoprotein of SARS-CoV-2. The biochip comprised gold nanostructure and γ-Fe_2_O_3_@Au core/shell nanoparticles. The γ-Fe_2_O_3_@Au nanoparticle is a complex particle, with a core of maghemite and a shell of gold. The γ-Fe_2_O_3_@Au nanoparticle has some special characteristics: the gold shell gives it good biocompatibility and the ability to conjugate with biomolecules. The maghemite core gives it magnetism and strong magneto-optical properties. Therefore, the γ-Fe_2_O_3_@Au nanoparticle is highly suitable for bio-applications [16, 17].

Current methods for identifying SARS-CoV-2 are based on the detection of the RNA, antibodies against spike glycoprotein and nucleocapsid phosphoprotein, or virus particles [18]. Isolation is presently the primary strategy used to prevent the spread of the epidemic. However, it is necessary to find an efficient way to determine infected individuals. Real-time PCR has been used to detect SARS-CoV-2 infections, with a limit of detection (LOD) from a few copies to a thousand copies of samples per milliliter [18, 19]. RT-PCR has the advantage of high sensitivity, but it takes several hours to complete the entire process. Its diagnostic accuracy is also affected by the preparation of the RNA [20]. Among many detection methods for SARS-CoV-2, RT-PCR is currently the most accuracy method. There are already many commercial RT-PCR kit for SARS-CoV-2 targeting different genes of the corona virus [21]. However, serological detection of antibodies targeting the viral envelope proteins is cheaper and faster but has poor sensitivity. Antibodies to the serum take weeks to develop, and the number of antibodies in a sample does not represent the original viral load of the patient [19, 22–24]. The detection of the spike glycoprotein of SARS-CoV-2 is a possible method to achieve a low-cost, high-speed, high-accuracy diagnosis. The spike glycoprotein is more specific than other proteins [25, 26]. Our research addressed the cost-effective and accurate diagnosis of SARS-CoV-2 on the basis of the detection of the spike glycoprotein.

## Materials and methods

### SARS-CoV-2 spike glycoprotein and antibody

The spike glycoprotein and its corresponding antibody (anti-S) were bought from Cusabio Technology LLC. The catalog number of the spike glycoprotein was CSB-MP3324GMY, and that of anti-S was CSB-RA33245A1GMY.

### Fabrication of MO CM Biochips

**Fig. 1:**
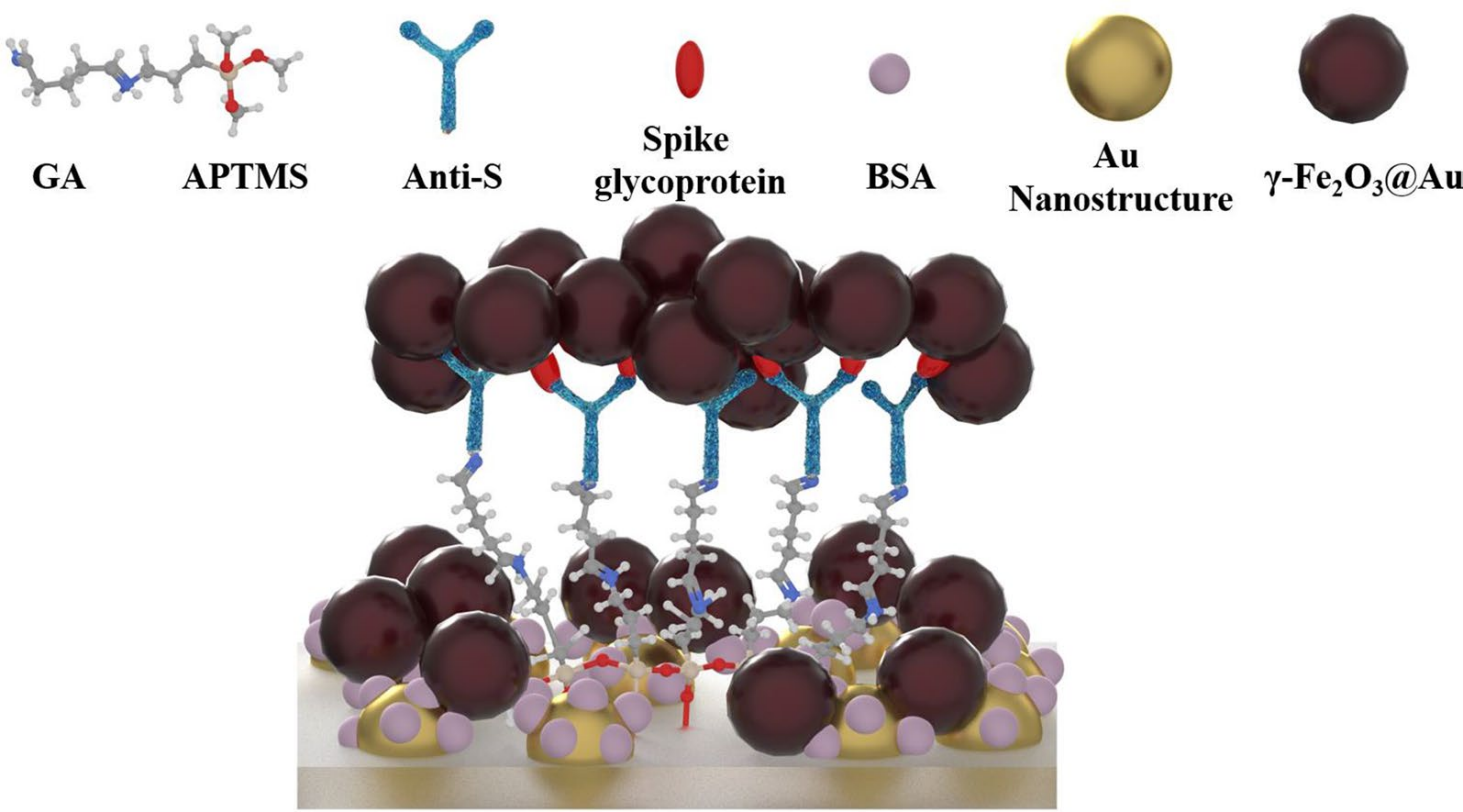
Schematic diagram of magneto-optical Cotton–Mouton biochip. (The representations of different particles are listed in the upper side of the figure. Besides, the red, blue, gray, light orange and white atoms in GA and APTMS represent oxygen, nitrogen, carbon, silicon and hydrogen molecules respectively)

Figure 1 shows the structure of the magneto-optical biochip. The chip is based on a 1.0 cm × 1.0 cm BK7 glass substrate covered with gold nanostructures that are fabricated by a gold thin film and annealing process. The gold thin film was deposited using an RF sputtering system. The thickness of the deposited gold thin film was 4 nm, and the annealing was conducted at 500 ℃ for 30 min in atmosphere. After annealing, the gold thin film breaks and shrinks to form the nanostructure. The gold nanostructure on the glass surface are the indicator to monitor the following surface functionalization processes. The chip was functionalized using (3-Aminopropyl)trimethoxysilane (APTMS) and glutaraldehyde (GA) [27]. The solvent for APTMS is the methanol. The chip was sequentially treated with APTMS (5% v/v, 40 μL) for 5 min, and GA (10% v/v, 40 μL) for 60 min. Next, the antibody of spike glycoprotein (anti-S, 10 μg/mL, 40 μL) was added and conjugated with GA. Finally, bovine serum albumin (BSA, 5% v/v, 40 μL) was used to block the remaining reaction sites. At the end of each fabrication step, the biochip was treated with phosphate-buffered saline (PBS, pH 7.4) and deionized (DI) water for washing. The biochip was then ready to detect the spike glycoprotein.

### MO CM measurement system

**Fig. 2:**
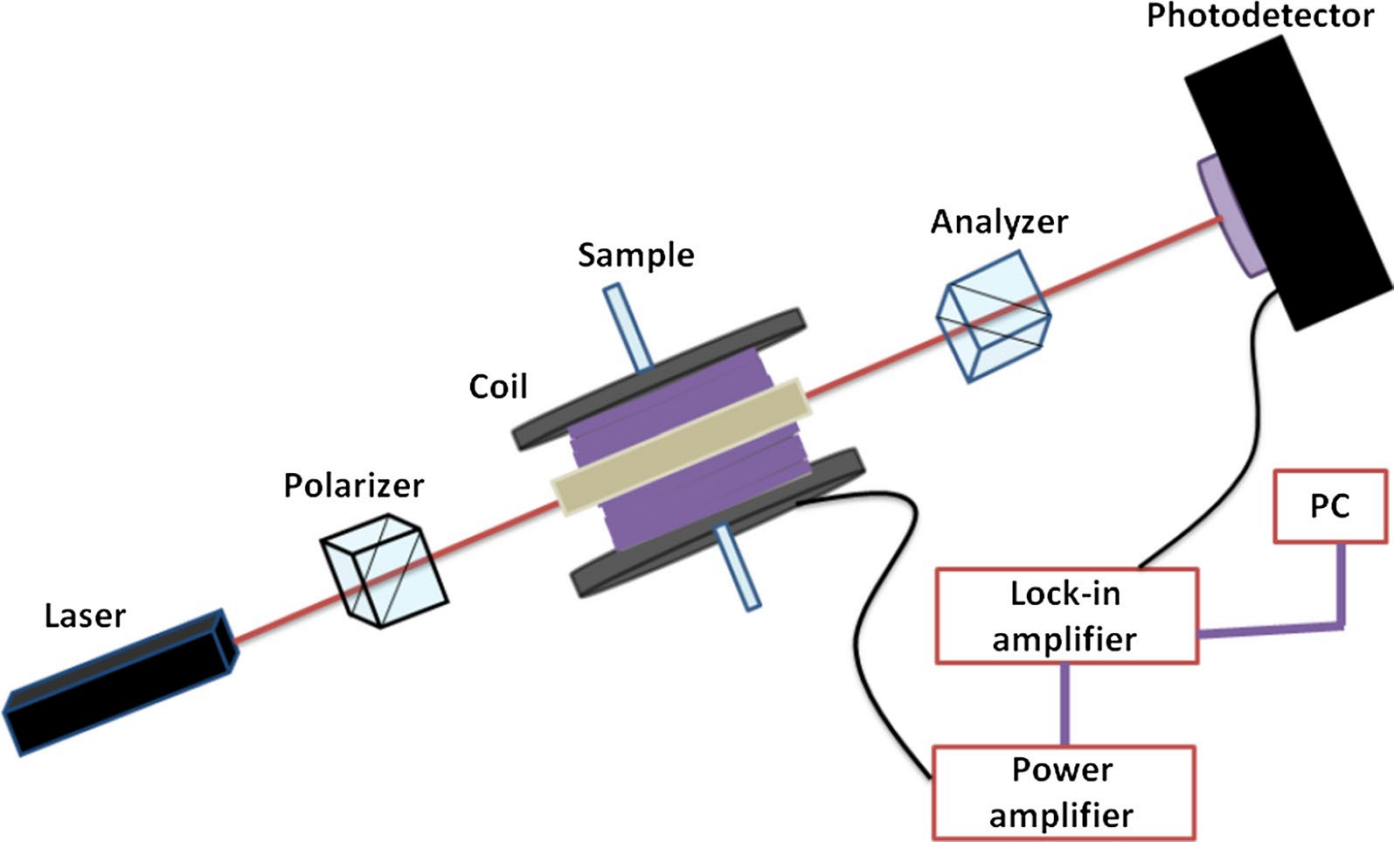
Schematic diagram of the MO CM effect measurement system

Figure 2 shows the schematic of the MO CM effect measurement system. The light source is a He–Ne laser with a wavelength of 633 nm. The analyzer rotates at an angle of *α* with respect to the polarizer. A homemade magnetic coil which are two solenoid coils divided by a plastic spacer so that there is a hole in the middle of the spacer for the light beam to go through is used to generate a magnetic field of 150 gauss, perpendicular to the direction of propagation of the light. The magnitude of the magnetic field generated by the homemade magnetic coil is verified and calibrated by the gauss meter. The lock-in amplifier generates a sine wave signal of 813 Hz to a power amplifier to drive the magnetic coil and records the signals using a PDA36A-CE (Thorlabs, Inc.) photodetector. The 813 Hz frequency of the lock-in amplifier was used in this work because it has the lowest background noise in our laboratory. When the magnetic field is turned off, the light intensity detected by the photodetector is *I*_*α*_ = *I*_*0*_cos^2^*α*. Assuming the MO CM effect induces a rotation *δ* of light polarization, it would induce a variance of light intensity Δ*I* = *I*_*0*_[cos^2^(*α* − *δ*) − cos^2^*α*]. Then, the rotation angle *δ* of the MO CM effect adapted to our system can be derived as [28]

1 $$\delta = \alpha -{\mathrm{cos}}^{-1}\left[{\left(1+\frac{\Delta I}{{I}_{0}}\right)}^{1/2}\mathrm{cos}\alpha \right]$$

In our system, *α* is set at 45°. If the applied magnetic field is sinusoidal with frequency ω, the light intensity detected by the photodetector becomes $I={I}_{0}+\Delta I$,where $\Delta I$ is the variation of the light intensity which depends on ω. Δ*I/I*_*0*_ can be obtained from the voltage *V*_*ac*_/*V*_*dc*_ analyzed by the lock-in amplifier, where *V*_*ac*_ and *V*_*dc*_ are the output voltages of the photodetector induced from Δ*I* and *I*_*0*_, respectively.

### γ-Fe_2_O_3_ nanoparticle synthesis

Initially, 5.40 g of FeCl_3_·6H_2_O and 1.98 g of FeCl_2_·4H_2_O powders were dissolved in 100.0 mL of DI water (2:1). Then, 30.0 mL of ammonia solution (2 M, pH = 10.0) was added into the ferric solution with vigorous stirring. Ferric ions were reduced to Fe_3_O_4_ magnetic nanoparticles, resulting in the formation of a black precipitate, which was washed thrice with DI water and collected by magnetic separation. The precipitate was re-dissolved in 100 mL HNO_3_ (0.1 M) and refluxed at 100 ℃ for 3 h. The Fe_3_O_4_ nanoparticles were subsequently oxidized to γ-Fe_2_O_3_ nanoparticles to form a reddish-brown colloidal solution. When the solution cooled to room temperature, 100.0 mL of 0.1 M tetra-methyl-ammonium hydroxide (TMAOH) was added, and the solution was centrifuged at 6000 rpm for 30 min to remove the supernatant. Finally, the precipitate was mixed with 200.0 mL of 0.1 M TMAOH and sonicated for 30 min to obtain a colloidal solution of γ-Fe_2_O_3_ nanoparticles for further use [4, 29].

### γ-Fe_2_O_3_@Au core/shell nanoparticle synthesis

First, 750 µL of γ-Fe_2_O_3_ colloidal solution was mixed with 100.0 mL DI water and sonicated for 15 min. Then, 3.0 mL of sodium citrate aqueous solution (0.1 M) was added and vigorously stirred for 1 h. In this step, the citrate ion (C_6_H_5_O_7_^3−^) replaced OH^−^ on the surface of the γ-Fe_2_O_3_ nanoparticles. Next, 200 µL of hydroxylammonium chloride (0.2 M) NH_2_OH·HCl and 500 µL of tetrachloroauric acid (1% v/v) were added for iterative gold seeding. This seeding process was repeated four times with an interval of 1 h between each iteration. This process allows Au^3+^ ions to reduce and form the Au shell on the surface of the γ-Fe_2_O_3_ nanoparticles. The color of the solution shifted from purple to deep garnet red as the Au layer was formed on the surface of γ-Fe_2_O_3_ nanoparticles. The colloidal solution of γ-Fe_2_O_3_@Au nanoparticles was magnetically centrifuged at 6000 rpm for 30 min and concentrated to 10.0 mL [4, 30].

### Assaying the spike glycoprotein of SARS-CoV-2

The assay process is as follows. Forty microliters of the detection sample are dropped on the biochip and incubated at 37 ℃ for 30 min. If the sample contains the spike glycoprotein, it reacts with the anti-S. Next, a wash step was performed to remove the unbound sample. The biochip is immersed into a Petri dish with a mixture solution of 1 mL PBS and 20 mL DI water for 1 min. Afterwards, the chip is taken out of the solution and drying with nitrogen. Then, 40 μL of reagent containing γ-Fe_2_O_3_@Au core/shell nanoparticles is dropped on the biochip and incubated at 20℃ for 15 min. Finally, the biochip is immersed in 20 mL DI water for washing out the unbound γ-Fe_2_O_3_@Au core/shell nanoparticles for 1 min. Again, a nitrogen flow is used to dry the biochip and then the chip can be taken to the optical system for MO CM effect measurement.

MO CM effect is a phenomenon of magnetic birefringence, which is relative to the sample birefringence Δ*n* = *n*_e_ ‒ *n*_o_, where *n*_e_ and *n*_o_ are the refractive indices of the extraordinary ray and the ordinary ray, respectively. The birefringence is a function of the wavelength *λ*, the magnetic field *B* and the Cotton–Mouton constant *C*^CM^ described by the relation [31]:

2 $$\Delta n = C^{{{\text{CM}}}} \lambda B^{{2}}$$

Moreover, the Cotton–Mouton constant may be written as follows [28, 31]:

3 $${C}^{\mathrm{CM}}=\frac{{\rho }^{N}}{30n{\varepsilon }_{0}\lambda }\left\{\frac{\Delta \chi }{{\mu }_{0}kT}+{\left(\frac{{\mu }_{m}}{kT}\right)}^{2}\right\},$$

where *ρ*^*N*^ is the volume concentration, *n* is the refractive index, *ε*_0_ is the permittivity of free space, *μ*_*m*_ is the permanent magnetic dipole moment, *Δχ* is the anisotropic magnetic susceptibility, *μ*_*0*_ is the permeability of free space, *k* is the Boltzmann constant and *T* is absolute temperature. It is obvious that the surface morphology of the arrangement of γ-Fe_2_O_3_@Au nanoparticles influences the anisotropic magnetic susceptibility *Δχ*. According to Eq. (2) and (3), the variation of *Δχ* induces the change of C^CM^ and hence causes the difference of MO CM effect. Different concentrations of anti-S cause different surface morphology of the chip and induce different MO CM effects. Therefore, the concentration of anti-S can be identified by measuring the MO CM effect.

Because of the gold shell of the γ-Fe_2_O_3_@Au nanoparticles, the γ-Fe_2_O_3_@Au nanoparticles can adhere to the protein. According to the work of Pakiari et al. [32], the interaction of amino acids with gold clusters is governed by two major bonding factors: (a) the anchoring N–Au, O–Au, and S–Au bonds and (b) the nonconventional N–H···Au and O–H···Au hydrogen bonds. Therefore, the γ-Fe_2_O_3_@Au nanoparticles can bind with BSA, Anti-S and spike glycoprotein. The spatial positions of γ-Fe_2_O_3_@Au nanoparticles which bind with different proteins are different. The existence of spike glycoprotein which conjugates with anti-S further changes the spatial position of γ-Fe_2_O_3_@Au nanoparticles. Therefore, different amount of spike glycoprotein can induce different morphology arrangement of γ-Fe_2_O_3_@Au nanoparticles which will induce the change of MO CM effect. In conclusion, the existence of the spike glycoprotein influences the surface morphology of the arrangement of γ-Fe_2_O_3_@Au nanoparticles and further induces the variation of the MO CM effects.

### Instruments for Biochips characterization

TEM and SEM were utilized to characterize the morphology of the biochip and the nanoparticles, respectively. The TEM used in this work is a high-resolution TEM (JEM-2010, JEOL Co. Ltd) with a maximum 200 kV accelerating voltage. SEM was conducted using a ZEISS Ultra Plus SEM with a maximum 30 kV accelerating voltage. Besides, hydrodynamic diameter and zeta potential of the nanoparticles are measured by the dynamic light scattering (SZ-100Z, HORIBA).

## Results and discussions

### Characterization of MO CM Biochips

**Fig. 3:**
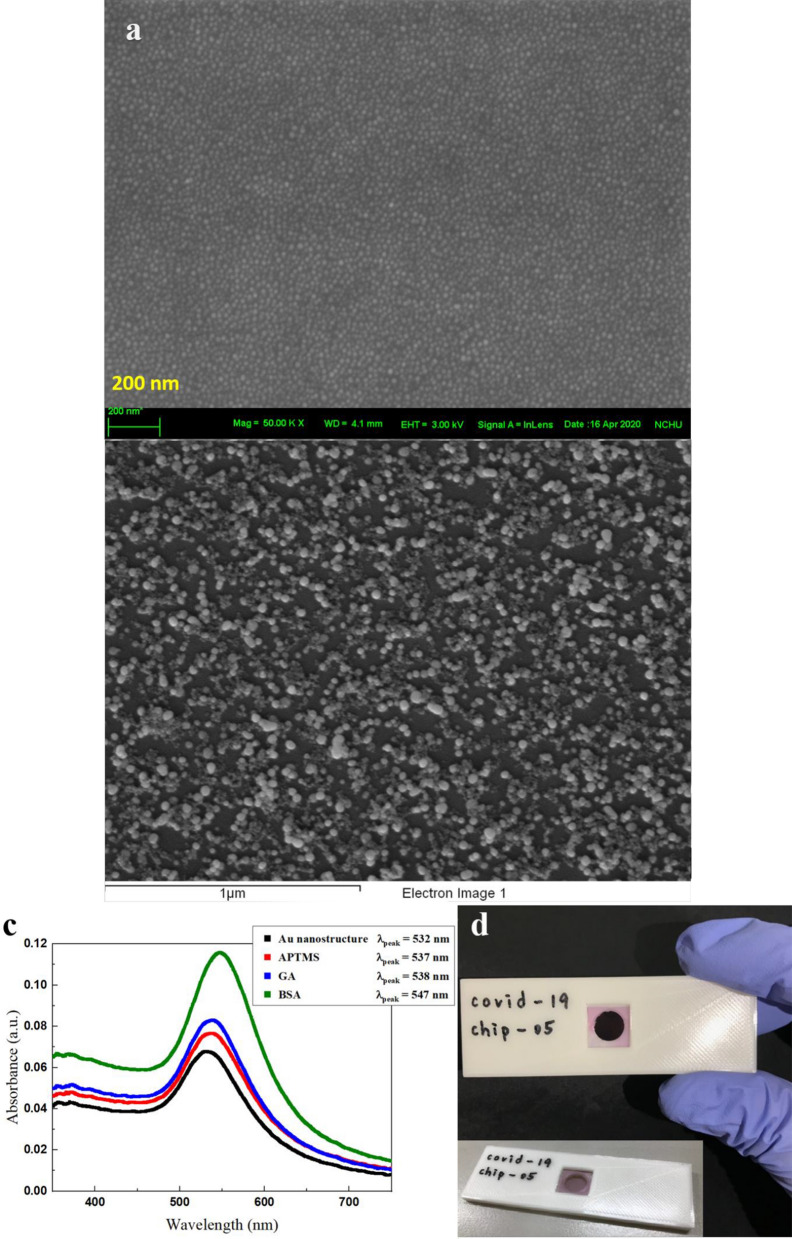
characterization of MO CM Biochips **a** SEM image of the gold nanostructure on a glass substrate (200 nm scale bar), **b** SEM image of the surface of the chip after adhering γ-Fe_2_O_3_@Au nanoparticles (1 μm scale bar), **c** Ultraviolet–visible spectrum of the biochip after the fabrication processes of Au nanostructure, APTMS, GA and BSA and **d** the photo of the biochip

Figure 3a shows a scanning electron microscopy (SEM) image of the gold nanostructure on the glass substrate. The average size of the nanostructure was approximately 20 nm. In the Fig. 3b, the surface morphology of the chip becomes rougher after adhering γ-Fe_2_O_3_@Au nanoparticles. Some bigger clusters of γ-Fe_2_O_3_@Au nanoparticles are formed and adhere to the chip surface. The EDX mapping of the Fig. 3b (Additional file 1: Figure S1) shows that there are elements carbon (C), iron (Fe), gold (Au) and oxygen (O) distributed on the biochip. These elements indicate the existence of the γ-Fe_2_O_3_@Au nanoparticles and the proteins on the surface of the biochip. Besides, the Ultraviolet–visible spectrum of the biochip in different stages of the fabrication are shown in the Fig. 3c. The absorption peaks of the biochip after the fabrication process of Au nanostructure, APTMS, GA and BSA are 532 nm, 537 nm, 538 nm and 547 nm, respectively. There are obvious redshifts of the absorption peaks after conjugating different molecular on the chip surface. The conjugating molecular changes the dielectric constant of the surroundings around the gold nanostructures on the chip and hence influences the localized surface plasmon resonance (LSPR) of the gold nanostructure. The redshift of LSPR indicates that that the fabrication is successful in different stages of the process. The APTMS will fill up the interspace between the gold nanostructure. Therefore, the modification of APTMS will induce the refractive index change of the surroundings around the gold nanostructure. The volume of the interspace with respect to the volume of the nanostructure is comparable. So, the refractive index change of the interspace can induce significant changes in the absorption intensity of nanostructure deposited on the glass substrate. It is the same that BSA introduction will induce the refractive index change of the surroundings around the gold nanostructure because BSA will adhere on the surface of gold nanostructure. Figure 3d shows the photo of the biochip of which the color is light pink due to the LSPR of the gold nanostructure.

### Characterization of γ-Fe_2_O_3_@Au

**Fig. 4:**
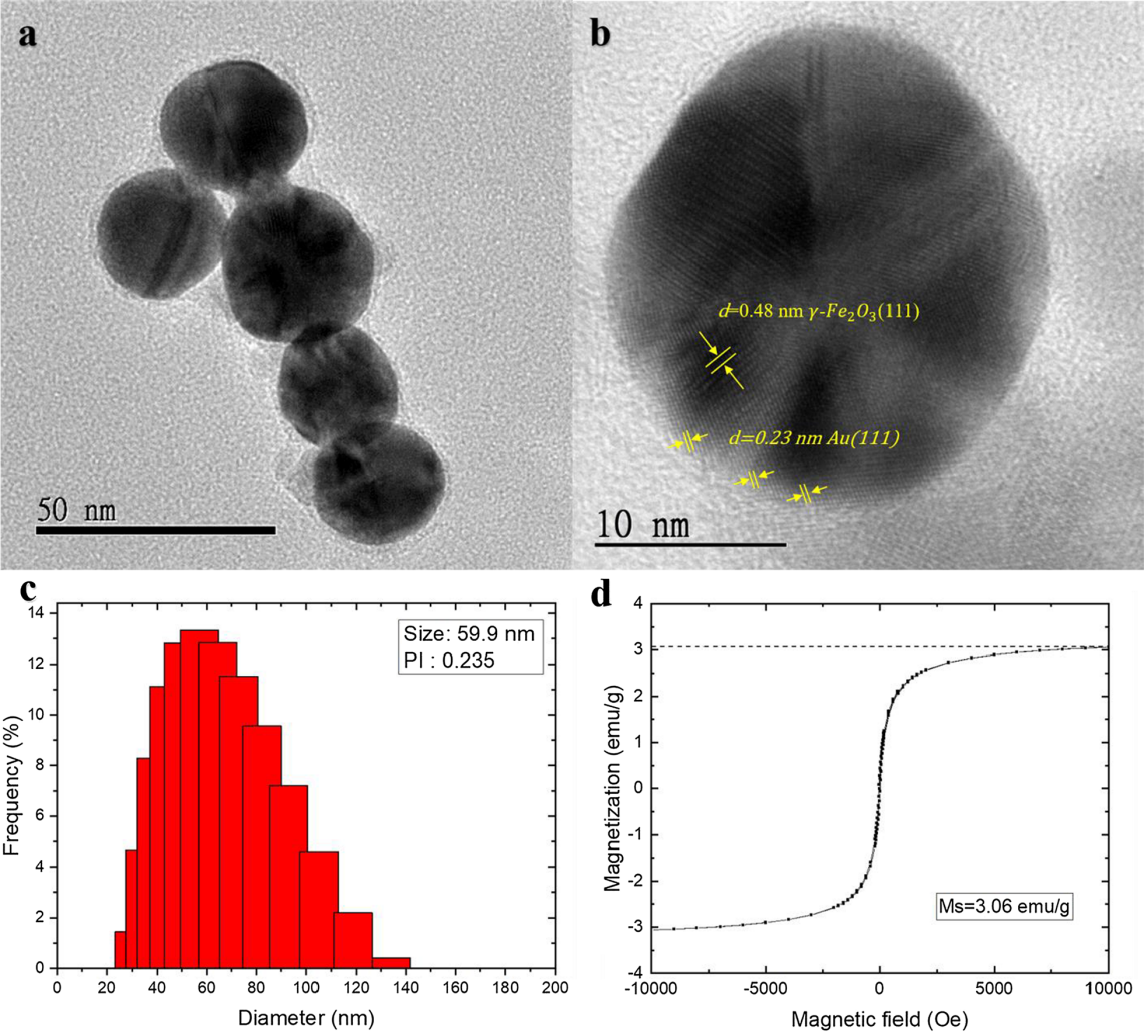
Characterization of γ-Fe_2_O_3_@Au nanoparticles **a** TEM image of γ-Fe_2_O_3_@Au nanoparticles (50 nm scale bar), **b** magnified TEM image of a γ-Fe_2_O_3_@Au nanoparticle lattice line of 0.48 nm γ-Fe_2_O_3_(111) and 0.23 nm Au (111) (10 nm scale bar), **c** distribution of the hydrodynamic diameter and **d** M-H curve of the γ-Fe_2_O_3_@Au nanoparticles

Figure 4a shows a transmission electron microscope (TEM) image of the γ-Fe_2_O_3_@Au nanoparticles. The particles are nearly spherical and have an average size of approximately 28 nm. A magnified TEM image (Fig. 4b) shows the crystal structure of the particle. It can be observed that the core structure of the particle is γ-Fe_2_O_3_, and the shell structure is gold. The macroscopic lattice structure of the γ-Fe_2_O_3_ and γ-Fe_2_O_3_@Au are verified by the X-ray diffraction analysis (Additional file 1: Figure S2). The XRD results indicate the good crystallinity of the γ-Fe_2_O_3_@Au nanoparticles. Besides, due to the heavy atom effect from the gold layer, the weakened peaks of the γ-Fe_2_O_3_ in the XRD of the γ-Fe_2_O_3_@Au also proves that the gold layer is coated on the surface of the γ-Fe_2_O_3_. Figure 4c shows the hydrodynamic sizes of the γ-Fe_2_O_3_@Au nanoparticle in the water, measured by the dynamic light scattering (DLS). The average hydrodynamic diameter of γ-Fe_2_O_3_@Au nanoparticle is 59.9 nm and the polydispersity index is 0.235. The zeta potential of γ-Fe_2_O_3_@Au nanoparticle is − 43.4 mV and it means that the γ-Fe_2_O_3_@Au nanoparticles can stably suspend in the water. The M-H curve of the solid γ-Fe_2_O_3_@Au nanoparticles obtained by MPMS Magnetometer (MPMS3, Quantum Design Inc.) is shown in Fig. 4d. The M-H curve is consistent with the Langevin function which can describe the magnetic response of MNP. It shows that the solid γ-Fe_2_O_3_@Au nanoparticles are approximately superparamagnetic and the saturation magnetization of the solid γ-Fe_2_O_3_@Au nanoparticles is 3.06 emu/g(γ-Fe_2_O_3_@Au). Besides, we use the co-precipitation method to synthesize MNPs, so the size of the produced MNPs may not be very uniform. The low coercivity (< 50 Oe) in the M-H curve may originate from the MNPs with the larger sizes.

### Detection of the spike glycoprotein of SARS-CoV-2 using MO CM biochips

**Fig. 5:**
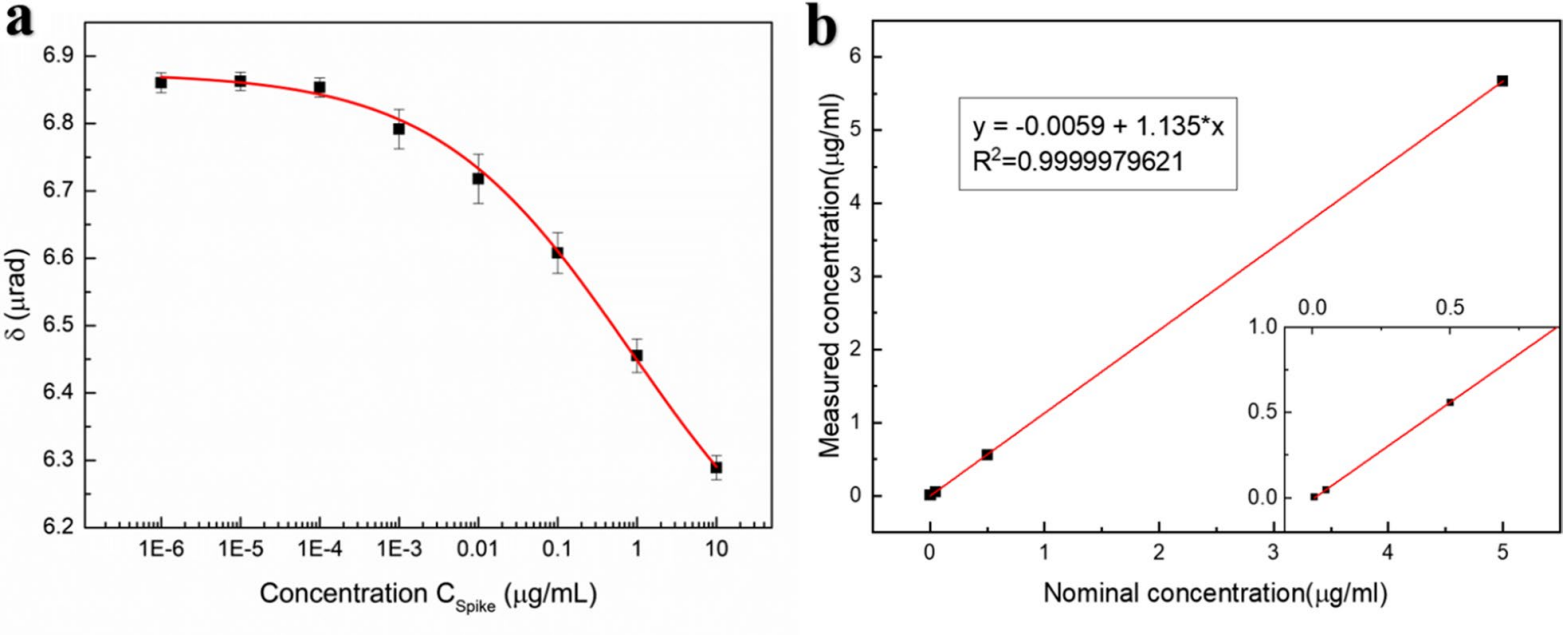
**a** Dependence of the concentration of spike glycoprotein C_spike_ (μg/ml) with respect to the CM rotation angle δ (μrad) form our MO CM biochips (the red line in the figure is the fitting curve of the logistic function), **b** results of the dilution linearity of the MO CM assay (insert: magnified image for low Nominal concentration)

Based on data from triplicate measurements, Fig. 5a shows the dependence of the concentration of spike glycoprotein with respect to the CM rotation angle under a 150 gauss magnetic field. The experiments were conducted in triplicates. In each experiment, the rotation angle was the average of a hundred readings from the sensor. From the obtained data, more spike glycoprotein induces larger changes in the CM rotation angle because more γ-Fe_2_O_3_@Au nanoparticles can adhere to the chip surface via conjugation with the spike glycoprotein. The dependence of the concentration of spike glycoprotein, *C*_*spike*_, and the CM rotation angle, *δ*, fits well (R^2^ = 0.998) to the logistic function

4 $$\delta =B+\frac{A-B}{1+{({C}_{spike}/{C}_{0})}^{p}}$$

where *A* = 6.88, *B* = 6.05, *p* = 0.356, and *C*_*0*_ = 0.833 are fitting parameters (Fig. 5a, solid red line). The logistic curve in Fig. 5a is also the standard curve used to estimate the concentration of spike glycoprotein. The LOD is calculated from the noise level (parameter *A*) and three standard deviations (3-σ criterion) [33]. The calculated LOD of *C*_*spike*_ is 0.27 ng/mL (3.4 pM). Besides, the right end of the curve in Fig. 5a has not yet reached saturation, the biochip can detect the spike glycoprotein at least 10 μg/mL which is the original concentration bought form the Cusabio Technology LLC.

We conducted a dilution linearity experiment to investigate the reliability of our assay method as shown in Fig. 5b. We prepared four samples with different concentrations of spike glycoprotein, via dilution with 50% glycerin recommended by the reconstitution guideline of the spike glycoprotein (CSB-MP3324GMY) from the supplier (CUSABIO). We measured these four samples and calculated their concentrations of spike glycoprotein using the standard curve in Fig. 5a. Table 1 shows the numerical results in Fig. 5b. Recovery is defined as the measured concentration divided by the nominal concentration. The recovery rates of the four samples were within the range of 80–120%, which is an acceptable recovery range. This result demonstrates that our assay method is reliable.

**Table 1:** Results of the dilution linearity of the MO CM assay

Nominal concentration (μg/mL)	Measured concentration (μg/mL)	Recovery (%)
5	5.67	113.4
0.5	0.56	112.0
0.05	0.047	94.0
0.005	0.0051	102.0

### Specificity of the biochips

**Fig. 6:**
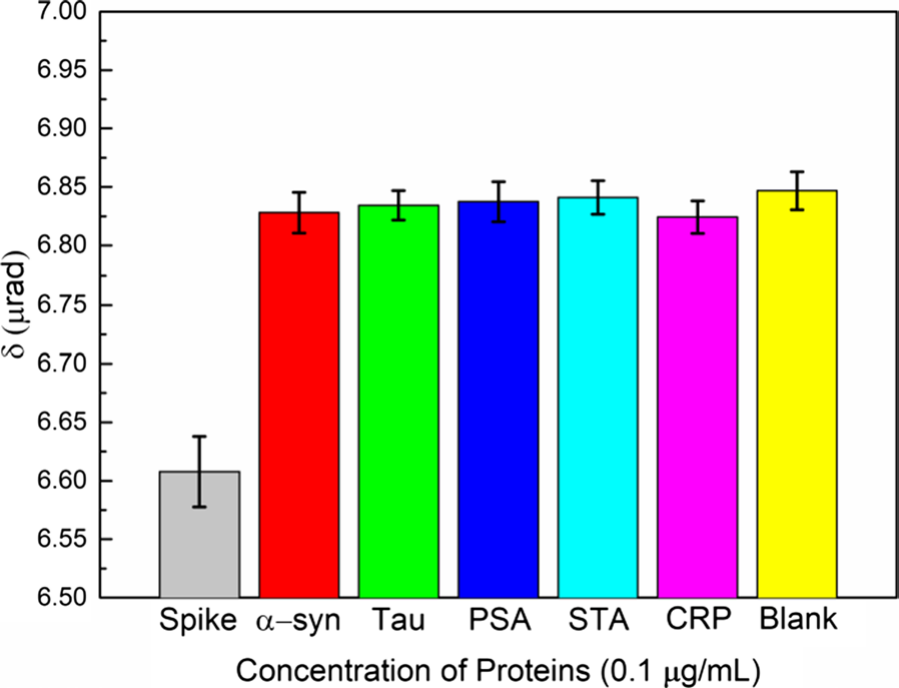
Results of the specificity experiment with all the concentrations of 0.1 μg/mL Spike glycoprotein, α-syn, Tau, PSA, STA, CRP proteins and blank sample

We also examined the specificity. Because the biochip is designed to assay SARS-CoV-2, it should be sensitive only to the spike glycoprotein. We prepared the samples with different proteins for the specificity test. The proteins used in the test are alpha synuclein (α-syn), Tau protein, Prostate Specific Antigen (PSA), streptavidin (STA), and C-reactive protein (CRP). Each sample was prepared with the same concentration of 0.1 μg/mL and measured in triplicate. The results in Fig. 6 show that the CM rotation angles of the 0.1 μg/mL α-syn, Tau protein, PSA, STA, and CRP assayed from our biochip are all around 6.83 μrad which is close to the result of the blank sample which is only DI water. However, the rotation angle of the 0.1 μg/mL spike glycoprotein is 6.60 μrad which can be clearly distinguished from the results of others proteins as shown in the Fig. 6. From this test, it can be clearly seen that only the spike glycoprotein can react with the biochip and induce changes in the MO CM effect. This result demonstrates that our biochip is highly specific to the spike glycoprotein of the SARS-CoV-2. A comparison of the LOD of the biosensors for the detection of the SARS-CoV-2 spike glycoprotein is listed in Table 2. The field-effect transistor (FET) biosensor reported by Seo et al. has an excellent performance for detecting the SARS-CoV-2 spike glycoprotein with an LOD of 1 fg/mL. A lateral flow point of care diagnostic device reported by Baker et al. can detect the SARS-CoV-2 spike glycoprotein with an LOD of 5 µg/mL in 30 min. Biosensors based on surface plasmonic such as functionalized terahertz plasmonic metasensors and surface enhanced Raman scattering (SERS)-based COVID-19 biosensor have an LOD of 4.2 fM and 0.77 fg/mL respectively. Our biochips based on the Cotton–Mouton effect of γ-Fe_2_O_3_@Au core/shell nanoparticles can detect SARS-CoV-2 spike glycoprotein with a relative good LOD of 0.27 ng/mL (3.4 pM) and short assay time (50 min) among other biosensors.

**Table 2:** Biosensors for the detection of the SARS-CoV-2 spike glycoprotein

Biosensor	Analyte (SARS-CoV-2)	LOD	Refs.
Graphene sheet field-effect transistor based biosensor	Spike glycoprotein	1 fg/mL	[34]
Electrochemical immunoassay	Spike glycoprotein	20 µg/mL	[35]
Bioelectric recognition assay based on membrane-engineered mammalian cells biosensor	Spike glycoprotein	1 fg/mL	[36]
Functionalized terahertz plasmonic metasensors	Spike glycoprotein	4.2 fM	[37]
5G-enabled fluorescence sensor	Spike glycoprotein	1.6 ng/mL	[38]
Surface enhanced Raman scattering (SERS)-based COVID-19 biosensor	Spike glycoprotein	0.77 fg/mL	[39]
Au-NPs@rPGO modified ITO electrode-based Surface enhanced Raman spectroscopy	Spike glycoprotein	39.5 fmol/L	[40]
AC magnetic response of Functionalized MNPs	Spike glycoprotein	0.084 nM	[41]
Opto-microfluidic sensing platform with gold nanospikes	Spike glycoprotein	0.08 ng/mL	[42]
A lateral flow point of care diagnostic device	Spike glycoprotein	5 µg/mL	[43]
A biochip based on the Cotton–Mouton effect of γ-Fe_2_O_3_@Au core/shell nanoparticles	Spike glycoprotein	0.27 ng/mL (3.4 pM)	This work

## Conclusions

We performed a proof-of-concept experiment and demonstrated an easy and quick assay method for the in vitro detection of the SARS-CoV-2. This assay method is achieved by using the MO CM effect and a biochip that is based on γ-Fe_2_O_3_@Au nanoparticles. Preliminary experimental results showed that the entire assay process takes approximately 50 min including 3 min for the CM rotation measurement, and the LOD of the spike glycoprotein is approximately 0.27 ng/mL (3.4 pM). Although the samples used in our work were not clinical specimens, we expect that the performance of our biochip can be comparable with the commercial ELISA kits for detecting the spike glycoprotein of SARS-CoV-2. The ELISA kits have an LOD of 2.7 ng/mL, and the assay time is longer [44]. Furthermore, the assay time and the LOD could be further improved with the optimization of our biochips. The noise equivalent power (NEP) of photodetector used in this work is about 0.593–29.1 pW/Hz^1/2^. Because the CM rotation is derived from the variance of the power intensity measured by the PD, it is reasonable to expect that the sensitivity of our system could be improved by a better PD with lower noise. In conclusion, the fabrication and operation of our biochip are simple, and it is easily modified to detect other diseases. Moreover, the design of our biochip is inexpensive. The cost of the biochip is low because it is made on the BK7 glass substrate and the amount of the related material usage is small. Each of the biochip costs less than $ 2 US dollars. Except for the optical measurement system, the biochip is easily portable. We think that our method is suitable for the quick assay at the outdoor screening station with an optical measurement system nearby. Besides, the biochip is designed to be disposable for getting the best assay result. We believe that our assay method is a promising tool for the rapid screening of patients in a pandemic, owing to the short assay time and the low LOD.

## Supplementary Information

**Additional file 1.** Additional information includes energy-dispersive X-ray spectroscopy (EDX) mapping images and EDX spectrum of the surface of the chip after adhering γ-Fe_2_O_3_@Au nanoparticles and X-ray diffraction analysis of the γ-Fe_2_O_3_ and γ-Fe_2_O_3_@Au nanoparticles.

## Data Availability

All data used to support the findings of this study are available from the corresponding author upon request.
