# Supplementary material for: A magneto-optical biochip for rapid assay based on the Cotton–Mouton effect of γ-Fe2O3@Au core/shell nanoparticles
Source: J Nanobiotechnology. 2021 Oct 1;19:301. doi: 10.1186/s12951-021-01030-z (PMC8485105; doi:10.1186/s12951-021-01030-z)
Supplement: Supplementary file 1 — Additional file 1. Additional information includes energy-dispersive X-ray spectroscopy (EDX) mapping images and EDX spectrum of the surface of the chip after adhering γ-Fe2O3@Au nanoparticles and X-ray diffraction analysis of the γ-Fe2O3 and γ-Fe2O3@Au nanoparticles. [file 12951_2021_1030_MOESM1_ESM.docx]

Additional File 1

Supplementary information for

**A magneto-optical biochip for rapid assay based on** **the** **Cotton–Mouton effect of γ-Fe_2_O_3_@Au core/shell nanoparticles**

Kuen-Lin Chen^1,2,*^, Zih-Yan Yang^2^ and Chin-Wei Lin^3^

^1^Institute of Nanoscience, National Chung Hsing University, Taichung, Taiwan
^2^Department of Physics, National Chung Hsing University, Taichung, Taiwan

^3^Graduate institute of applied physics, National Taiwan University, Taipei, Taiwan

**
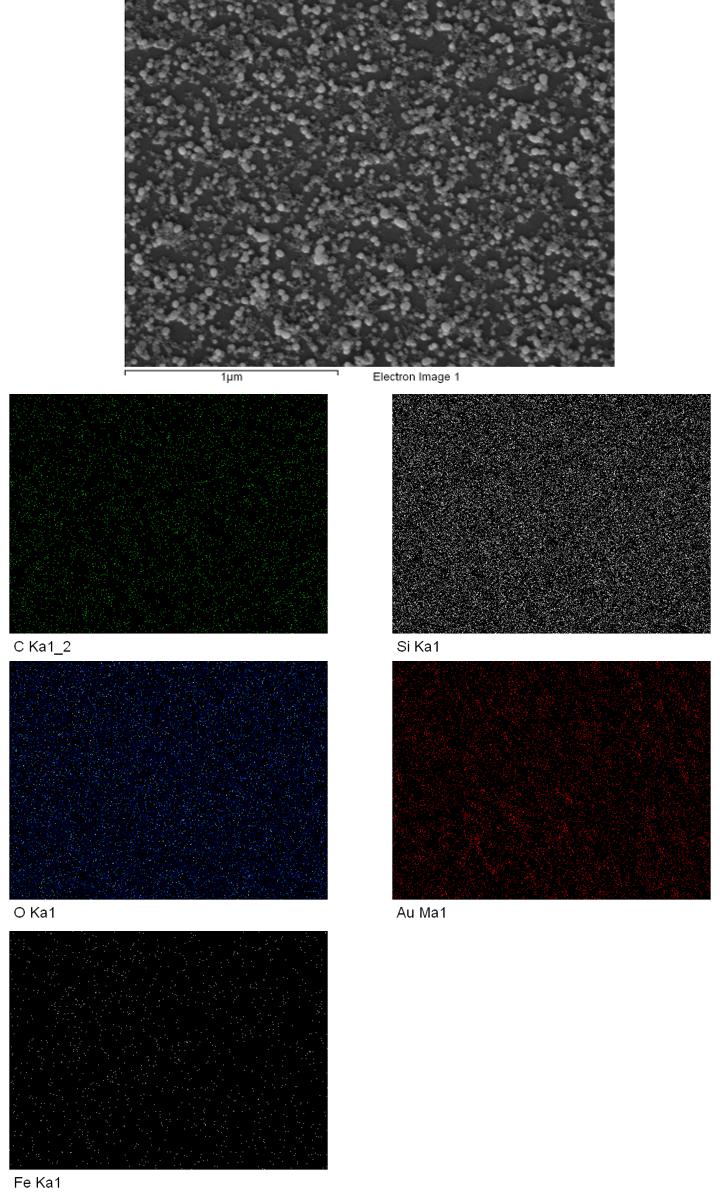
**

**
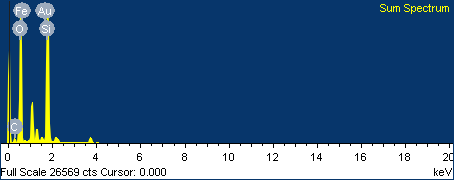
**

**Figure S1. Energy-dispersive X-ray spectroscopy (EDX**) **mapping images of the surface of the chip after adhering γ-Fe_2_O_3_@Au nanoparticles and the corresponding EDX spectrum.**

The figure S1 shows the Energy-dispersive X-ray spectroscopy (EDX) mapping images of the surface of the chip after adhering γ-Fe_2_O_3_@Au nanoparticles. The element carbon (C) may come from the APTMS, GA, BSA, anti-S and spike protein. On the other hand, iron (Fe) comes from γ-Fe_2_O_3_, silicon (Si) is from the glass substrate and gold (Au) comes from the gold layer of γ-Fe_2_O_3_@Au and Au nanostructure on the glass. There are some other small peaks in the EDX spectrum, which may come from the impurities in the glass substrate.


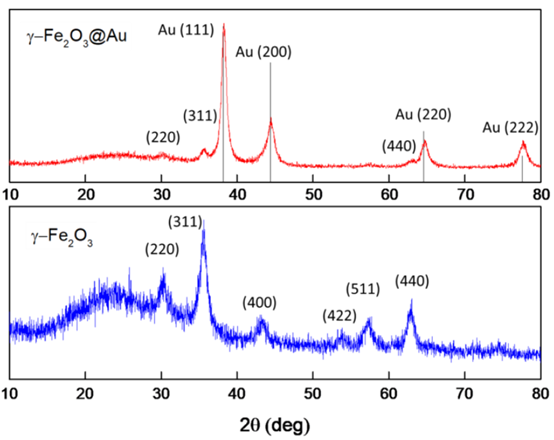


**Figure S2. X-ray diffraction analysis of the γ-Fe_2_O_3_ and γ-Fe_2_O_3_@Au.**

The figure S2 shows the X-ray diffraction (XRD) analysis of the γ-Fe_2_O_3_ and γ-Fe_2_O_3_@Au. The sharp and strong diffraction peaks of γ-Fe_2_O_3_ appear at 2θ = 30°, 35°, 43°, 50°, 57°, 62° corresponding to the faces of γ-Fe_2_O_3_ (220), (311), (400), (422), (511), and (440) respectively [1-2]. The diffraction peaks show at 2θ = 38°, 44° and 64° corresponding to the faces of Au (111), (200), (220), and (222) [3]. Some peaks of γ-Fe_2_O_3_ are almost disappeared in the XRD of γ-Fe_2_O_3_@Au. Only the small peaks of γ-Fe_2_O_3_ (220), (311), and (440) can be seen in the XRD of γ-Fe_2_O_3_@Au. The weakened peaks of the γ-Fe_2_O_3_^­^ in the XRD of the γ-Fe_2_O_3_@Au originates from the heavy atom effect of the gold [4]. If the gold does not cover the γ-Fe_2_O_3_, it will form individual Au particles elsewhere and cause the existence of both strong peaks of γ-Fe_2_O_3_ and gold in the XRD. The weakened peaks of the γ-Fe_2_O_3_^­^ in the XRD of the γ-Fe_2_O_3_@Au proves that the gold layer is coated on the surface of the γ-Fe_2_O_3_ to form γ-Fe_2_O_3_@Au core/shell structure.

1. C. Caizer, I. Hrianca. Dynamic magnetization of γ-Fe2O3nanoparticles isolatedin an SiO2amorphous matrix. THE EUROPEAN PHYSICAL JOURNAL B. 2003;391–400.
2. S. Hei, Y. Jin, F. Zhang. Fabrication of 𝛾-Fe_2_O_3_ Nanoparticles by Solid-State Thermolysis of a Metal-Organic Framework, MIL-100(Fe), for Heavy Metal Ions Removal. Journal of Chemistry. 2014;3.
3. G. Geng, P. Chen et al. Sheet like gold nanostructures/graphene oxide composites via a one-pot green fabrication protocol and their interesting two-stage catalytic behaviors. RSC Adv. 2017;7:51838.
4. Z. C. Xu, Y. L. Hou, S. H. Sun, Magnetic core/shell Fe_3_O_4_/Au and Fe_3_O_4_/Au/Ag nanoparticles with tunable plasmonic properties. J. Am. Chem. Soc. 2007;129:8698-8699.
